# Hydrophilic composite nanoparticles of wheat gluten protein and carboxymethyl cellulose by pH cycle and its emulsification properties for loading curcumin^☆^

**DOI:** 10.1016/j.fochx.2025.103096

**Published:** 2025-09-29

**Authors:** Pengren Zou, Celia Costas, Susan Jyakhwo, Yihao Luo, Li Luo, Zhaojun Wei, Paz Otero

**Affiliations:** a Universidade de Vigo, Nutrition and Bromatology Group, Department of Analytical Chemistry and Food Science, Faculty of Science, 32004 Ourense, Spain; b International Institute “Solution Chemistry of Advanced Materials and Technologies”, ITMO University, 191002 Saint Petersburg, Russia; c Animal Science Department, College of Agriculture and Animal Husbandry, Qinghai University, Ningda Road 251, 810016 Xining, China; d Xuyong Langjiu Oriental Glass Co.,Ltd, 646000 Luzhou, Sichuan, China; e School of Biological Science and Engineering, Specialty Food Nutrition and Health Innovation Team of Ningxia Hui Autonomous Region, North Minzu University, Yinchuan 750021, China

**Keywords:** Solubility, Nanoparticles, Co-assembly, Crosslinking, Interactions, Digestion

## Abstract

Wheat gluten proteins (WPs) constitute cross-linked dough due to its physochemical properties. However, its application is limited because of its low solubility. Nutraceuticals' encapsulation inside nanoparticles to improve their bioaccessibility presents some challenges. This study aims to construct WPs-carboxymethyl cellulose (CMC) nanoparticles (WCM) by pH cycling, as well as to characterize their physicochemical properties, and assess their applicability as nanocarriers. Alkalization exposed WPs binding sites, allowing co-assembly with CMC by hydrophobic and electrostatic interactions. CMC prevents protein refolding during neutralization. Its fiber network made WCM nanoparticles' structure more orderly with moderately increased crystallinity, favoring their emulsifying function. WCM 2:1 nanoparticles with particle size of 832.40 nm and polydispersity index of 0.38 could be loaded with curcumin, increasing its *in vitro* bioaccessibility up to 64.68 %. This study provides more insight into pH-driven *co*-assembly between hydrophobic proteins and polysaccharides, contributing to their development as innovative materials and functional factor delivery carriers.

## Highlights

• pH cycling exposes specific wheat gluten residues.
• Carboxymethyl cellulose stabilizes the unfolded state of proteins.
• WCM 2:1 nanoparticles displayed optimal particle size and uniform distribution.
• WCM nanoparticle emulsions encapsulate curcumin to improve its bioaccesibility.

## Graphical abstract

**Figure:**
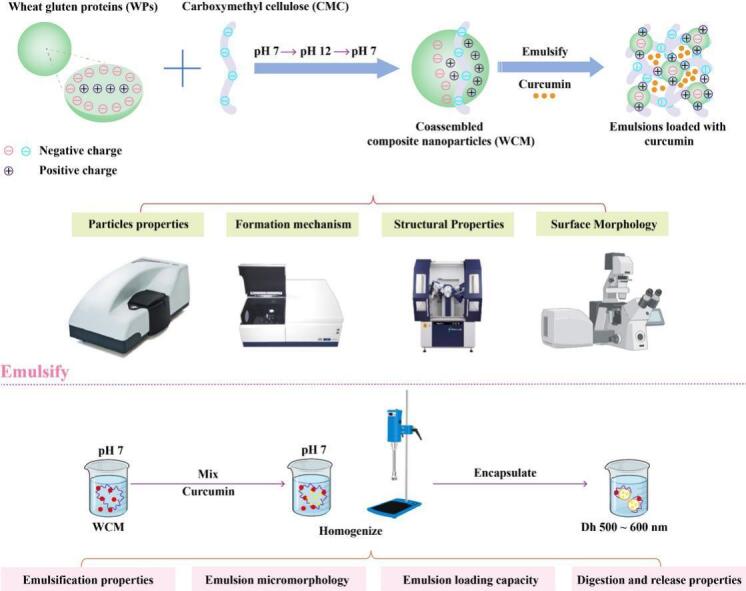

## 1 Introduction

Wheat gluten, a cost-effective and nutrient-rich plant protein, exhibits emulsification, gelation, viscoelasticity, and film-forming properties, rendering it valuable for diverse applications (Hu et al., 2022). The unique physicochemical characteristics of wheat gluten proteins (WPs) arise from their high proportion of uncharged and hydrophobic amino acid residues, which promote crosslinking and aggregation, thereby limiting their solubility in aqueous solvents (Xiao, Zou, Hu, Zhu, & Wei, 2023). WPs mainly consist on glutenin and gliadin, which are predominantly utilized in dough-based products, however with limited exploration in emulsion systems. Recent studies have shown that modulating external conditions can induce conformational changes in these proteins, enabling the formation of multifunctional assembled structures (Solomonov, Kozell, & Shimanovich, 2024). Accordingly, WPs can be engineered into various self-assembled forms, including films, hydrogels, micelles, vesicles, and nanoparticles, making it feasible for applications such as biosensors, coatings, emulsions, controlled release of bioactive compounds, and the development of functional foods (Tang, 2021; Zou et al., 2025).

Protein self-assembly, driven by reversible or irreversible aggregation of its fragments, is primarily mediated by chemical reactions or non-covalent interactions, including hydrogen bonding, Van der Waals forces, and π-π stacking (Mohammadi, Hayati, Jafari, Karimi, & Gutierrez, 2019). These structural transformations are triggered by altering environmental conditions such as ionic strength, mechanical forces, pH, and temperature. In recent years, pH cycling has emerged as a simple, effective, and environmentally friendly technique for inducing structural assembly in insoluble plant proteins to enhance their solubility (Qing et al., 2022). However, self-assembled protein structures alone often lack the functionality required for multifaceted applications. Several studies have demonstrated that pH cycling promotes a transition in WPs to a molten globule state, characterized by intact secondary structure units similar to those of native proteins (Gong et al., 2024; Xiong et al., 2023; Zou et al., 2022). This partially unfolded state facilitates interactions between WPs and other molecules, enabling the formation of novel hydrophilic structures. Thus, WPs can be co-assembled and crosslinked with ligands, such as polysaccharides, lipids, or active compounds (*e.g.*, organic acids, flavonoids, phenolic compounds), to form complexes with enhanced properties and novel functionalities (Cameselle et al., 2025; Ren et al., 2024; Zou et al., 2025). For instance, He, Wang, Feng, Chen, and Wang (2020) reported that inducing the molten globule state in WPs at pH 12, followed by co-solubilization with soy proteins and pH adjustment to 7, enabled co-assembly into edible hydrophilic colloids. Furthermore, *co*-assembly of globular proteins with ionic polysaccharides, facilitated by various techniques, yields electrostatic complexes such as protein-polysaccharide nanoparticles, which have shown promising applications. This has spurred significant interest in leveraging biologically biocompatible plant-derived proteins and polysaccharides to develop co-assembled structures for controlled delivery of bioactive compounds and nutrients.

Carboxymethyl cellulose (CMC), a plant-derived polysaccharide, possesses a negatively charged surface that facilitates electrostatic interactions with positively charged protein molecules, enabling crosslinking and the formation of hydrophilic complexes. The abundance of hydrophilic groups in CMC promotes co-crosslinking with WPs through intramolecular or intermolecular hydrogen bonding, which plays a critical role in the co-assembly of nanocomplexes. For example, Fu et al. (2019) utilized pH cycling to construct wheat gluten nanoparticle-xanthan gum complexes that stabilized Pickering emulsions, effectively encapsulating β-carotene and enhancing its bioaccessibility and stability. Similarly, Li et al. (2023) demonstrated that co-assembled wheat glutenin-CMC colloidal carriers improved the stability of vitamin D against long-term storage and ultraviolet radiation, while also enabling controlled release during gastrointestinal digestion. Due to the complementary structure and function of WPs and CMC, their co-assembly is expected to produce functional nanocomposites with tunable three-dimensional network structures, solving the problems of low protein solubility and poor emulsification performance of polysaccharides when used separately. Driven by the pH cycling method, plant-derived WPs and CMC are co-assembled to synthesize safe and edible complex nanomaterials, enabling them to transition from simple applications to more diverse ones, and even exhibit unexpectedly superior functions in certain aspects.

Although previous studies have assessed pH-driven methods to induce *co*-assembly of proteins and polysaccharides to form nanocomposites, scarce data has been reported about the interaction patterns and mechanisms between both of them during the alkalization and neutralization stages. Therefore, this study aims to construct hydrophilic nanocomposites with excellent performance through pH cycling-induced co-assembly, which can be applied to emulsion systems for the encapsulation and delivery of hydrophobic active substances. Moreover, the folding behavior of WPs induced by pH cycling along with its patterns of co-assembly with CMC during the neutralization process were evaluated. Their structural features and formation mechanisms were also studied to provide more insight into its suitable application as insoluble-bioactive molecules' carriers. Additionally, the emulsification properties and stability of the nanocomposites were assessed to explore their practical applications. Finally, the potential of these nanocomplex emulsions as encapsulation carriers and deli*v*ery systems for hydrophobic curcumin was investigated.

## 2 Materials and methods

### 2.1 Samples and reagents

Wheat gluten was obtained from Anhui Ante Foods Co., Ltd. Carboxymethyl cellulose was purchased from Shanghai Aladdin Reagent Co., Ltd. Curcumin, pepsin, and lipase were obtained from Shanghai Yuanye Bio-Technology Co., Ltd. All other chemicals, of analytical grade, were purchased from Sinopharm Chemical Reagent Co., Ltd. (Shanghai, China).

### 2.2 WPs extraction

WPs were extracted following the method described by Zou et al. (2022). Wheat gluten was dispersed in deionized water at a 1:10 (*w*/*v*) ratio. The pH of the dispersion was adjusted to 12.0 using 3 M NaOH and stirred magnetically at 750 rpm for 2 h. The mixture was centrifuged at 8000 rpm for 10 min to remo*v*e impurities, including starch. The supernatant was collected, and its pH was adjusted to 4.0 with 6 M HCl. After centrifugation at 8000 rpm for 10 min, the resulting precipitate was collected and subsequently freeze-dried to obtain purified WPs.

### 2.3 Preparation of WPs-CMC composite nanoparticles

Composite nanoparticles of WPs and CMC were prepared using a modified pH cycling method adapted from Li and Zhong (2021). As controls, 1 g of WPs or CMC was individually dissolved in 200 mL of distilled water with stirring until fully dispersed. For composite nanoparticles, WPs were combined with varying amounts of CMC at WPs: CMC mass ratios (*w*/w) of 10:1, 5:1, 2:1, 1:1, and 1:2, maintaining a constant WPs concentration of 0.5 % (*w*/*v*). The pH of each mixture was adjusted to 12.0 using 3 M NaOH and stirred at 60 °C for 30 min (750 rpm). Subsequently, the pH was gradually adjusted to 7.0 with 6 M HCl. The resulting solution was centrifuged at 8000 rpm for 10 min, and the supernatant was collected to obtain WPs-CMC composite nanoparticle solutions. These were designated as WCM 10:1, WCM 5:1, WCM 2:1, WCM 1:1, and WCM 1:2, corresponding to the respective WPs: CMC mass ratios.

### 2.4 Proteins solubility

Protein concentrations in all samples were determined using a Coomassie Brilliant Blue reagent kit of beyotime company (China, Shanghai). A standard curve was generated by measuring the absorbance of bovine serum albumin (BSA) solutions (0.125–1.5 mg/mL) at 595 nm. Solutions of WPs-CMC composite nanoparticles were adjusted to pH 12.0 with 3 M NaOH to dissociate the complexes, and their protein concentrations were subsequently measured. Protein solubility was calculated using the following equation:

(1) $$\;\mathrm{Solubility}\;\left(\%\right)=C_{1}/C_{O}\times 100$$

Where, C_1_ represents the protein concentration measured in the sample supernatant (mg/mL), and C_0_ is the initial protein concentration of 5.0 mg/mL (1 g WPs in 200 mL of water).

### 2.5 Particles characterizations

The average hydrodynamic diameter (Dh), polydispersity index (PDI) and zeta potential of WPs, CMC, and WPs-CMC composite nanoparticle solutions (WCM 10:1, WCM 5:1, WCM 2:1, WCM 1:1, and WCM 1:2) were determined by dynamic light scattering (DLS) at 25 °C using a Nano-ZS90 Laser Particle Sizer. Additionally, a solution containing 1 g of WPs and 0.5 g of CMC (2:1 mass ratio) in 200 mL of deionized water was prepared, and the pH was adjusted to 12.0 as described in Section 2.3. The zeta potential was measured during the neutralization process at pH values of 12.0, 11.0, 10.0, 9.0, 8.0, and 7.0.

### 2.6 Fluorescence spectroscopy

Endogenous fluorescence spectra of WPs, CMC, and WPs-CMC composite nanoparticle solutions (WCM 10:1, WCM 5:1, and WCM 2:1) were recorded using an F97Pro fluorescence spectrophotometer (Shanghai Lengguang, China). Each solution was excited at 280 nm, and emission spectra were collected from 300 to 600 nm. The CMC solution consisted of 1 g dissolved in 200 mL of deionized water. To investigate the interactions driving co-assembly, 1 g of WPs and 0.5 g of CMC (2:1 mass ratio) were dissolved in 200 mL of deionized water, and inhibitors—thiourea, sodium chloride (NaCl), or sodium dodecyl sulfonate (SDS)—were added to achieve a final concentration of 10 mmol/L. These solutions underwent the pH cycling method described in Section 2.3, and their endogenous fluorescence spectra were measured as described above to assess the roles of hydrogen bonding, electrostatic, and hydrophobic interactions in WPs-CMC co-assembly.

For exogenous fluorescence measurements, 4 mL of WPs, WCM 10:1, WCM 5:1, or WCM 2:1 solutions were mixed with 10 μL of 8 mmol/L 8-anilino-1-naphthalenesulfonic acid (ANS). Fluorescence spectra were recorded from 460 to 560 nm at an excitation wavelength of 390 nm. Additionally, a solution of 1 g WPs and 0.5 g CMC (2:1 mass ratio) in 200 mL of deionized water was prepared and adjusted to pH 12.0 as per Section 2.3. Exogenous fluorescence spectra were measured at pH values of 12.0, 11.0, 10.0, 9.0, 8.0, and 7.0 during the neutralization step to evaluate changes in surface hydrophobicity.

### 2.7 Structure characterization

#### 2.7.1 Surface hydrophobicity (Ho)

Solutions of WPs, WCM 10:1, WCM 5:1, and WCM 2:1 were diluted to 1, 0.75, 0.50, 0.25, and 0.125 times their original concentrations, and the corresponding protein concentrations were determined as described in Section 2.4. For each diluted solution, 4 mL of each diluted solution was mixed with 10 μL of 8 mmol/L ANS. Fluorescence intensity was measured at an excitation wavelength of 390 nm and an emission wavelength of 484 nm using an F97Pro fluorescence spectrophotometer (Shanghai Lengguang, China). Surface hydrophobicity (*Ho*) was calculated as the initial slope of the fluorescence intensity *versus* protein concentration curve. Additionally, the WCM 2:1 solutions prepared at pH 12.0, 11.0, 10.0, 9.0, 8.0, and 7.0 (as described in Section 2.6) were analyzed for *Ho* using the same method to assess changes in surface hydrophobicity during pH neutralization.

#### 2.7.2 Fourier transform infrared spectroscopy (FTIR)

Solutions of WPs, CMC, WCM10:1, WCM5:1, and WCM2:1 were freeze-dried and analyzed using a Nicolet 6700 FTIR spectrometer (Thermo Electric Corporation, MA, USA) equipped with attenuated total reflectance (ATR) mode. Spectra were recorded over a wavenumber range of 650–4000 cm^−1^ with a resolution of 4 cm^−1^, averaging 32 scans per sample.

#### 2.7.3 X-ray diffraction (XRD)

The freeze-dried samples from Section 2.7.2 (WPs, CMC, WCM 10:1, WCM 5:1, and WCM 2:1) were analyzed using a D/MAX 2500 *V* X-ray diffractometer (Rigaku Corporation, Tokyo, Japan) equipped with Cu-Kα radiation (λ = 1.5406 Å). Diffraction patterns were collected over a 2θ range of 5° to 60° at a scan rate of 2°/min. Data were processed and analyzed using MDI Jade 6.0 software.

### 2.8 Morphology characterization

#### 2.8.1 Transmission electron microscopy (TEM)

Solutions of WPs, CMC, WCM 10:1, WCM 5:1, WCM 2:1, and WCM 1:1 were diluted to appropriate concentrations and deposited onto carbon-coated copper grids. The samples were subjected to con*v*entional negative staining and air-dried. Morphological analysis was performed using a JEM1400FLASH transmission electron microscope (Hitachi, Japan) at an accelerating voltage of 80 kV.

#### 2.8.2 Atomic force microscope (AFM)

Solutions of WPs, CMC, WCM 10:1, WCM 5:1, WCM 2:1, and WCM 1:1 were diluted to suitable concentrations, and drop-cast onto smooth flat mica substrates. The samples were dried at room temperature and *v*isualized using a Dimension Fast Scan atomic force microscope (Bruker, Germany) operated at a scanning frequency of 1 Hz.

### 2.9 Emulsion characterization

Solutions of WPs, CMC, WCM 10:1, WCM 5:1, WCM 2:1, and WCM 1:1, prepared as described in Section 2.3, were mixed with linseed oil at a 9:1 (*v*/v) ratio and stirred thoroughly to disperse the oil droplets. Emulsions were formed by shearing the mixtures using a D-160 high-speed homogenizer (Dalong, China) at 15,000 rpm for 3 min.

#### 2.9.1 Emulsifying activity

Immediately after emulsion formation and after 10 min, 0.05 mL of each emulsion was withdrawn from the bottom of the container and mixed with 5 mL of 0.1 % (*w*/*v*) SDS solution, followed by incubation for 5 min. The absorbance of each sample was measured at 500 nm using a UV–visible spectrophotometer. 10 mL of each WPs, CMC, WCM 10:1, WCM 5:1, WCM 2:1, and WCM1:1 solution before emulsified was freeze-dried to obtain their corresponding solid mass concentrations (C). Emulsifying acti*v*ity index (EAI) and emulsion stability index (ESI) were calculated referring the method of Wang, Li, Sun, and Zhang (2023) as the following equations:

(2) $$\mathrm{EAI}\;\left(m^{2}/g\right)=\left(2\times 2.303\times A_{0}\times N\times 10^{-4}\right)/\left(\varphi \times L\times C\right)$$

(3) $$\mathrm{ESI}\;\left(\min\;\right)=A_{0}/\left(A_{0}-A_{10}\right)\times 10$$

Where, A_0_ and A_10_ are the absorbance values of the emulsion at 0 and 10 min, respectively; N is the dilution factor 100; φ is the oil phase fraction 0.10; L is the cuvette path length 0.01 m; and C is the solid sample concentration before emulsification, mg/mL.

#### 2.9.2 Degree of flocculation

The average particle size of each emulsion was measured using dynamic light scattering with deionized water and 0.1 % (*w*/*v*) SDS as dispersants. The degree of flocculation (F) was calculated referring the method of Alade, Mahmoud, Al Shehri, and Sultan (2021) as the following equation:

(4) $$F\;\left(\%\right)=\left(d-d_{\mathrm{SDS}}\right)/d_{\mathrm{SDS}}\times 100$$

Where, d and d_SDS_ are the average particle diameters (nm) of the emulsion measured with water and SDS as dispersants, respectively.

#### 2.9.3 Interfacial protein content

Freshly prepared emulsions were centrifuged at 10,000 rpm for 10 min, and the lower aqueous phase was collected and filtered (0.22 μm). The protein concentration in the aqueous phase was measured as described in Section 2.4. The interfacial protein content (A) of the emulsion was calculated referring the method of Lin et al. (2025) as the following equation:

(5) $$A\;\left(\%\right)=\left(C-C_{s}\right)/C\times 100$$

Where, C is the total protein concentration in the emulsion (mg/mL), and C_s_ is the unadsorbed protein concentration in the aqueous phase (mg/mL).

#### 2.9.4 Emulsion stability analysis

The stability of the WCM 2:1 emulsion was evaluated under various conditions. Emulsions were subjected to water-bath treatments at 25 °C, 50 °C, 70 °C, and 90 °C for 30 min, pH adjustments to 4.0, 5.0, 6.0, 7.0, and 8.0, and addition of NaCl at concentrations of 0, 50, 100, 200, and 400 mM. After 24 h at 25 °C, their Dh and PDI were measured (as detailed in Section 2.5). Additionally, freshly prepared WCM 2:1 emulsions were stored in containers at 25 °C in the absence of light for 1, 5, 10, 20, and 30 days, and their Dh and PDI were determined to assess flocculation and aggregation.

### 2.10 Microscopic characterization of emulsions

#### 2.10.1 Optical microscopy

Emulsions prepared from WPs, CMC, WCM 10:1, WCM 5:1, and WCM 2:1 were examined using a TCS SP8 laser confocal scanning microscope (Leica, Germany) to assess their morphology and droplet shape.

#### 2.10.2 Fluorescence microscopy

To elucidate the emulsion structure, the oil phase and protein components were stained separately. A dye solution containing 300 μL of Nile Blue (0.1 %, *w*/*v*) and 300 μL of Nile Red (0.01 %, w/v) was mixed with 10 mL of each emulsion. The mixture was incubated in a dark room for at least 30 min to ensure thorough staining. Images were acquired using Ar/Kr and He/Ne dual-channel laser modes, for oils and proteins, with excitation wavelengths of 488 nm (for Nile Red) and 633 nm (for Nile Blue), respectively.

### 2.11 Emulsion loading and digestion characterization

Curcumin-loaded emulsions were prepared following the method described in Section 2.9. Curcumin was dissolved in linseed oil (1 mg/mL) to serve as the oil phase, while WCM 10:1, WCM 5:1, and WCM 2:1 solutions were used as the aqueous phase.

#### 2.11.1 Retention rate

Curcumin standard solutions (1–10 μg/mL) in anhydrous ethanol were prepared to construct a standard curve. All emulsion loaded with curcumin were preserved at 4 °C, subsequently their curcumin retention was analyzed after 1, 5, 10, 20, and 30 days. To determine the curcumin content in the loaded emulsion, 1 mL of the emulsion was mixed with 9 mL of anhydrous ethanol, vigorously shaken, and centrifuged at 8000 rpm for 10 min. The supernatant's and standard curve's absorbance was measured at 430 nm using a UV–visible spectrophotometer, and the curcumin concentration was calculated from the standard curve. The retention rate (RT) was calculated using the following equation:

(6) $$\mathrm{RT}\;\left(\%\right)=C_{t}/C_{0}\times 100$$

Where, C_0_ and C_t_ are the curcumin concentrations (mg/mL) in the loaded emulsion at day 0 and at time (t), respectively.

#### 2.11.2 *In vitro* digestion

An *in vitro* digestion model, adapted from Jo, Ban, Goh, and Choi (2021), was used to evaluate the digestive properties of curcumin-loaded WCM 10:1, WCM 5:1, and WCM 2:1 emulsions, with a linseed oil emulsion containing an equivalent amount of curcumin as the control. Simulated gastric fluid (SGF) was prepared with 3.2 mg/mL pepsin, 2 mg/mL NaCl, and 7 mL/L concentrated HCl (36–38 %). Simulated intestinal fluid (SIF) contained 24 mg/mL lipase, 36.7 mg/mL CaCl₂·2H₂O, 218.7 mg/mL NaCl, and 54 mg/mL bile salt.

**Gastric Digestion**: 5 mL aliquot of the loaded emulsion was mixed with 20 mL of SGF (preheated to 37 °C for 5 min), and the pH (<2.5) was adjusted to 2.5 using 1 M NaOH. The mixture was incubated at 37 °C with shaking (100 rpm) in a thermostatic water bath for 2 h. Samples were collected at 30, 60, 90, and 120 min.

**Intestinal Digestion**: The pH of the gastric-digested emulsion was adjusted to 7.0 with 2 M NaOH, and 20 mL of preheated SIF (37 °C for 5 min) was added. The mixture was incubated at 37 °C with shaking (100 rpm) for 2 h, intermittently measuring every 30 min and maintaining pH 7.0 with 1 M NaOH or HCI. Samples were collected at 30, 60, 90, and 120 min.

Each digested sample was centrifuged at 10,000 rpm for 30 min, separating into an upper oil phase, a middle clarified micellar phase containing curcumin, and a bottom insoluble precipitate, as described by Liu, Wang, He, Cheng, and Ma (2020). A 200 μL aliquot of the micellar phase was mixed with 1800 μL of anhydrous ethanol, centrifuged at 8000 rpm for 10 min, and the supernatant was analyzed for curcumin content (C_t_) using UV–visible spectrophotometry at 430 nm. The release rate was calculated using the following equation:

(7) $$\mathrm{Release rate}\;\left(\%\right)=C_{t}/C_{0}\times 100$$

Where, C_0_ and C_t_ represent the curcumin content (mg/mL) in the initial emulsion and in the digest at the corresponding collection time, respectively.

To assess bioaccessibility, the final digestion mixture was centrifuged at 10,000 rpm for 30 min, and the clarified micellar phase was collected to measure its curcumin content (C_a_). Bioaccessibility (M) was calculated using the following equations:

(8) $$M\;\left(\%\right)=C_{a}/C_{0}\times 100$$

### 2.12 Statistical analysis

All experiments were performed in triplicate. Data were processed and analyzed using Origin, SPSS, and Adobe Illustrator software. Statistical significance was determined using one-way analysis of variance (ANOVA) plus Dunn's Multiple Comparison Test, with a significance level set at *p* < 0.05.

## 3 Results and discussion

### 3.1 Particles properties

**Fig. 1:**
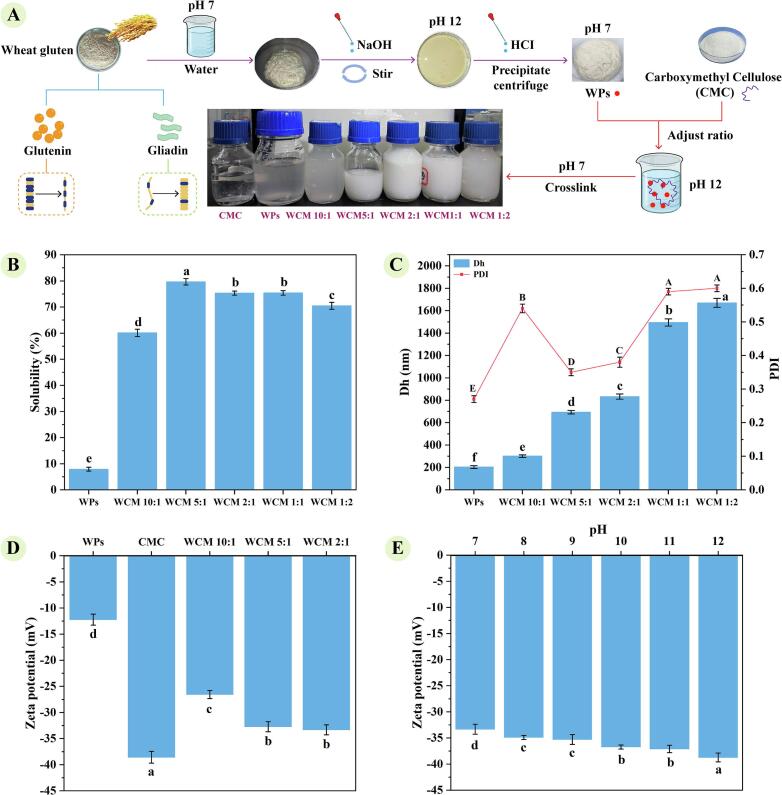
Preparation and characterization of composite nanoparticles (WCM) from wheat gluten protein (WPs) and carboxymethyl cellulose (CMC); (A) Schematic diagram of preparation process; (B) Solubility; (C) Particle size (Dh) and Polydispersity index (PDI); (D) Zeta potential; (E) Zeta potential during the preparation of WCM 2:1 nanoparticles from pH 12 to 7. The a-e indicated the mean values were significantly different (*p* < 0.05).

As the pH of the mixed solution was adjusted from 12.0 to 7.0, the cationic regions of WPs increasingly interacted with the anionic groups of CMC *via* noncovalent interactions, facilitating the formation of WCM nanocomplexes (Fig. 1-A). Although the pH 12 improved the dispersion of WPs in water, significant sedimentation persisted as the pH adjusted back to neutrality according to the previous report of He, Wang, et al. (2020). As shown in Fig. 1-B, WPs exhibited low protein solubility in water (7.92 %). However, pH cycling induced WPs folding, self-assembly, and co-assembly with CMC, forming dispersible WCM nanoparticles that increased protein solubility up to 79.67 %. Protein solubility peaked at a WPs: CMC ratio of 5:1, but further increasing in CMC content led to a decline, likely due to repulsion between anionic polysaccharide groups and steric hindrance (Yang, Wang, Yang, & Liu, 2024). This aligns with findings by He et al. (2020), who reported significantly improved protein solubility (up to 92.1 %) of wheat gluten co-assembled with an appropriate shellac through pH cycling method.

As shown in Fig. 1-C, the average particle size of WCM nanoparticles increased with higher WPs: CMC ratios, particularly for WCM 1:1 and WCM 1:2, which exceeded 1000 nm. The PDI followed a similar trend, with WCM 5:1 and WCM 2:1 exhibiting lower values (Fig. 1-C), indicating greater stability and uniformity. The particle size range of WCM nanoparticles (301.13–832.40 nm) is comparable to that of β-lactoglobulin-arabic gum (250–400 nm), making them unsuitable for biopharmaceutical systems (<200 nm), but ideal for food emulsions (Cao et al., 2021).Therefore, WCM 10:1, WCM 5:1 and WCM 2:1 nanocomplexes were chosen for subsequent studies due to their relatively small particle size as well as better stability. The absolute zeta potential of WCM nanoparticles was significantly lower than that of CMC alone (38.57 mV), but higher than that of WPs alone (12.23 mV), confirming electrostatic interactions between WPs and CMC (Fig. 1-D). Notably, zeta potential values increased along with CMC content, suggesting a dominant influence of the polysaccharide compared to proteins in these systems. The average particle size of WCM 2:1 nanoparticles was greater than those of WCM 5:1, but the PDI values (WCM 2:1 0.38, WCM 5:1 0.35) and zeta potential (WCM 2:1–33.33 mV, WCM 5:1–32.73 mV) were similar and within a stable range. Combined with the result that WCM 2:1 nanoparticles protein solubility (75.34 %) was lower than that of WCM 5:1 (79.67 %), indicating that WCM 2:1 contained a relatively lower proportion of WPs and a higher percentage of CMC. Therefore, in WCM 2:1 nanoparticles, WPs are relatively more effective to co-assemble with an appropriate amount of CMC through pH cycling and form stable bonds. During pH adjustment from 12.0 to 7.0, the zeta potential measurements of the WCM 2:1 solution revealed a gradual decrease in its value, further evidencing electrostatic complexation between WPs and CMC (Fig. 1-E). These findings corroborate that pH cycling enhances WPs solubility and enables *co*-assembly with polysaccharides to form stable nanocomplexes suitable for applications in emulsion-based food systems.

### 3.2 Formation mechanism of WCM

**Fig. 2:**
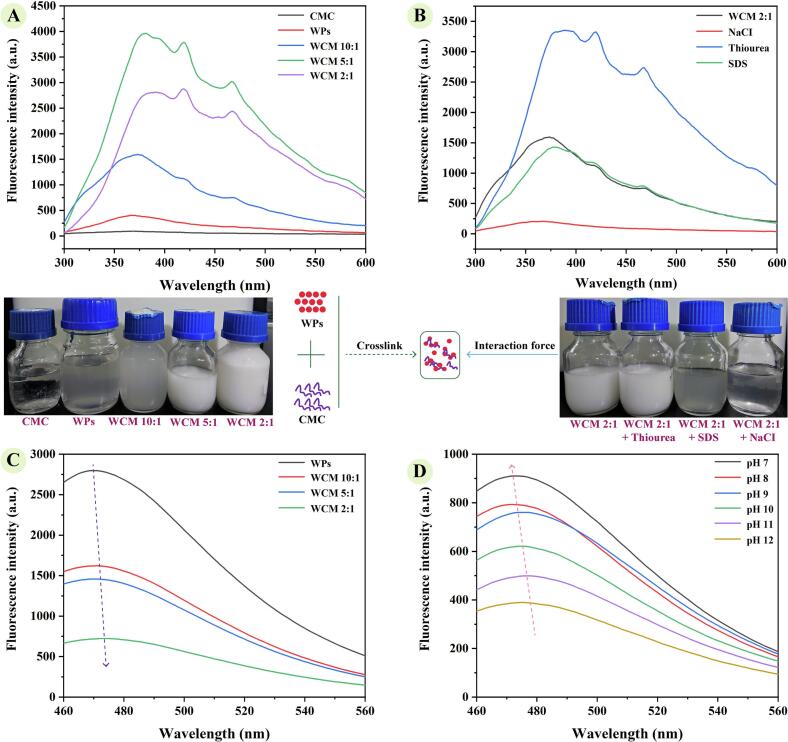
Revealing the formation mechanism of WCM by fluorescence spectroscopy; (A) Endogenous fluorescence emission of samples at pH 7.0; (B) Endogenous fluorescence emission of WCM 2:1 added with 10 mM SDS, NaCl, and thiourea; (C) Exogenous fluorescence emission of 8-Anilino-1-naphthalenesulfonic acid (ANS) bound to samples; (D) Exogenous fluorescence emission of WCM 2:1 at pH 7–12. a.u. means arbitrary units.

As shown in Fig. 2-A, the fluorescence intensity of the WPs solution was low, but it increased with the addition of CMC, peaking at WCM 5:1. The isoelectric point of WPs ranges from 6.0 to 8.0 (Zou, Hu, Ni, et al., 2022), suggesting that alkalization can make them unfold. Adjusting the pH of the WPs solution from 7.0 to 12.0 unfolded the protein's tertiary structure, exposing aromatic amino acids from the hydrophobic core to the aqueous environment. The presence of CMC inhibited protein refolding during pH neutralization (from 12.0 to 7.0), maintaining the protein's unfolded state during their co-assembly. Due to the long molecular chains and branched structure of CMC, it can prevent proteins from forming compact folded structures through steric hindrance, maintaining a relatively unfolded state. The crucial reason is that the interaction between WPs and CMC during neutralization affects the solubility and folding state of proteins. Additionally, the fluorescence spectra of WCM nanoparticle solutions exhibited a red shift, indicating a transition from a hydrophobic to a hydrophilic protein structure. These observations suggest that pH cycling promotes the exposure of specific amino acid residues within WPs subfractions, and co-assembly with CMC stabilizes this conformation, enhancing the fluorescence intensity of WCM nanoparticles.

The co-assembly of WPs and CMC is predominantly driven by non-covalent interactions. To investigate these, thiourea, SDS, and NaCl were used as inhibitors of hydrogen bonding, hydrophobic interactions, and electrostatic interactions, respectively (He, Chen, et al., 2020). The fluorescence intensity of the WCM 2:1 solution increased with thiourea, whereas SDS and NaCl reduced it (Fig. 2-B). This indicates that hydrophobic and electrostatic interactions are the primary forces governing their co-assembly. Since the negatively charged CMC binds to the cationic groups on the surface of WPs through electrostatic attraction. This binding shields the charges on the surface of proteins, reducing their electrostatic repulsive forces, thereby inhibiting protein folding. For hydrophobic interactions, CMC can regulate hydrophobic cross-linking within WPs, altering the local conformation of proteins, which stabilizes the spatial structure of WCM nanoparticles. The isoelectric point (pI) value of WPs (6–8) is higher than that of soy protein isolate (pI≤4.5), and it contains lower levels of charged amino acids. Soy protein isolate and CMC co-assemble into complexes mainly prepared at acidic pH, and they have relatively stronger electrostatic interactions than WPs (Feng et al., 2024). Compared with many globular proteins, the hydrophobicity of WPs is relatively strong, enabling hydrophobic interactions to play a key role in the co-assembly process with CMC and contributing to the stability of the composite nanoparticles (Wang et al., 2024).

Binding of ANS to hydrophobic protein domains enhances fluorescence intensity, enabling evaluation of protein conformation. As shown in Fig. 2-C, the exogenous fluorescence intensity of WCM solutions decreased with increasing CMC content, accompanied by a red shift in the peak emission wavelength. This indicates that CMC inhibits protein refolding during pH neutralization from 12.0 to 7.0, promoting a hydrophilic conformation through co-assembly with WPs. Furthermore, WCM 2:1nanoparticle was specifically selected for analysis due to the fact that it displayed a strong shift toward more hydrophilic conformation state. The exogenous fluorescence intensity of the WCM 2:1 solution was lowest at pH 12.0 (Fig. 2-D), suggesting a molten globule state in which hydrophobic regions are internalized, resulting in a hydrophilic exterior. As the pH decreased to 7.0, the fluorescence intensity of the WCM 2:1 solution gradually increased, indicating protein refolding and a shift toward a hydrophobic conformation. This is consistent with previously reported results for hydrophobic Zein (Wan et al., 2023), where pH cycling alters the folding behavior of the protein, exposing its binding site and enabling it to co-assemble with CMC. These findings confirm that pH cycling induces protein unfolding, while co-assembly with CMC stabilizes a hydrophilic conformation in WCM nanoparticles.

### 3.3 Structural properties

**Fig. 3:**
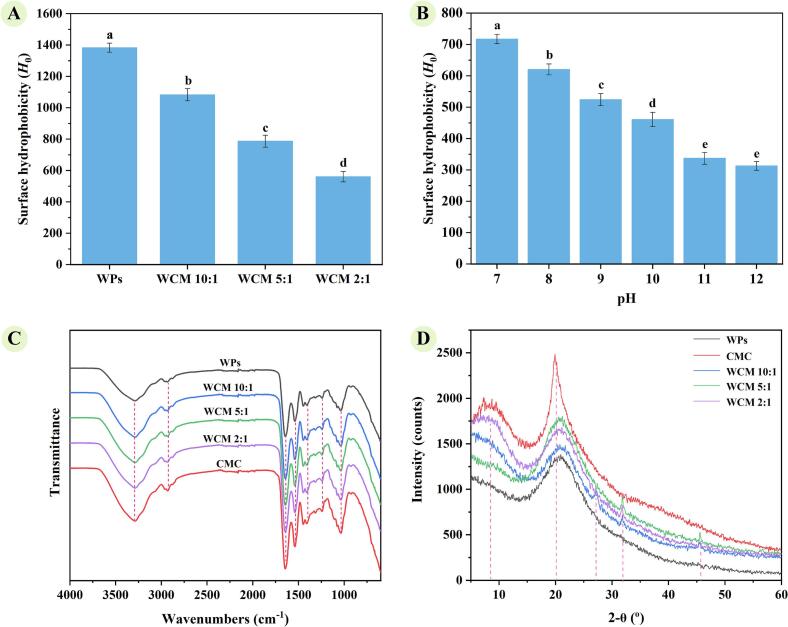
Characterization of structural properties; (A) Surface hydrophobicity of samples at pH 7; (B) Surface hydrophobicity of WCM 2:1 at pH 7–12; (C) FTIR spectrum; (D) X-Ray spectrum. The a-e indicated the mean values were significantly different (p < 0.05).

Protein structure is closely linked to solubility, and conformational analysis provides insights into dissolution behavior in aqueous environments. Surface hydrophobicity primarily depends on the exposure of aromatic and aliphatic amino acid residues, reflecting the extent of hydrophobic groups in contact with the polar solvent (Hu et al., 2022). As shown in Fig. 3-A, the *Ho* of WCM nanoparticle solutions decreased from 1382.83 for WPs to 560.26 for WCM2:1 as the WPs: CMC ratio decreased, indicating reduced exposure of hydrophobic regions and enhanced hydrophilicity in the co-assembled structure. Additionally, the *Ho* of WCM 2:1 increased from 312.59 at pH 12.0 to 717.37 at pH 7.0 (Fig. 3-B), suggesting that co-assembly with CMC is accompanied by pH-dependent structural changes in WPs. Thus, pH cycling modulates hydrophobic sites in WPs, while the incorporation of CMC stabilizes a hydrophilic three-dimensional structure, preventing excessive exposure of WPs' hydrophobic regions in the aqueous environment (Xiao et al., 2023).

FTIR spectroscopy (Fig. 3-C) revealed similarities among WPs, CMC, and WCM nanoparticle spectra. This is attributed to the fact that WPs and CMC both contain abundant hydroxyl, carboxyl/amide, and ether groups, and the vibration modes of these chemical bonds cause them to produce similar absorption peaks. Peaks around 3250 cm^−1^ correspond to the stretching vibrations of O—H, -NH₂, and -CONH₂ groups related to hydrogen bonds (Mohammadi, Gutiérrez, Hayati, Mohammadi, & Rezaei, 2019). WCM nanoparticles showed more broadened and intensified peaks with increasing CMC content when compared to WPs. In addition, they showed a slight shift to the right, tending to be aligned with the CMC spectrum. This change suggests stronger hydrogen bonding in WCM nanoparticles, contributing to their enhanced hydrophilic structure. Peaks in the 2900–2800 cm^−1^ range is indicative of C—H stretching, and the 1400–1700 cm^−1^ region encompasses amide bands and carboxyl group, whereas the 1000–1200 cm^−1^ fragment is related with C—O groups (Lian et al., 2025). They exhibited similarities in their peak in these areas, consistent with the results of egg white protein-CMC assembly previously reported by Li et al. (2025). Theoretically, proteins contain characteristic amide II bands (∼1540 cm^−1^, N—H bending and C—N stretching), while CMC has more pronounced symmetric and antisymmetric -COOH vibration peaks (∼1410 cm^−1^ and ∼ 1600 cm^−1^). In the 2800–3000 cm^−1^ range, CMC primarily exhibits C—H stretching, while the protein side chains of WPs may result in a different spectral pattern in this region. Therefore, these regions may be used to distinguish the spectral differences between WPs and CMC. Due to the influence of instrument resolution and samples, their spectra were very similar here and did not show obvious differences.

XRD analysis (Fig. 3-D) showed distinct spectra for WCM nanoparticles compared to WPs and CMC, with peaks at 7°, 20°, 28°, 32°, and 45° (2θ). The main feature of WPs is a broad amorphous halo ring, typically at 2θ ≈ 20–22°, which reflects the protein's weakly ordered nature (Li et al., 2023). CMC is predominantly amorphous, but commonly exhibits two broad peaks: one at 2θ ≈ 10°, resulting from short-range ordering due to side chains/substitution degree, and another at 2θ ≈ 19–21°, arising from short-range secondary ordering caused by residual stacking of cellulose main chains (Lawal et al., 2023). CMC displayed higher peak intensities at 20° (2θ), whereas WPs showed broader peaks. As the WPs: CMC ratio decreased, WCM nanoparticles exhibited a peak at 7° (2θ) consistent with CMC and a peak at 20° (2θ) similar to WPs. This suggests that WPs interact with CMC (hydrogen bonding, ion pairing, and phase separation), changing their peak position, width, and relative intensity. Higher peak intensities reveal greater crystallinity, while broader peaks (larger full width at half maximum) suggest smaller crystal sizes (Bhatnagar et al., 2019). With increasing CMC proportion, the WCM nanoparticles narrowed at 20° (2θ), indicating enhanced local ordering. In addition, WCM nanoparticles exhibited changes in relative intensity at 2θ ≈ 10°, which was influenced by variation in the stacking arrangement of CMC segments. Moderate increases in crystallinity are beneficial for WCM nanoparticles in forming a denser and more resilient interfacial film in emulsions, reducing their permeability and enhancing their mechanical stress resistance. This suggests that WCM 2:1 nanoparticles have a relatively more orderly and compact binging structure, which is conducive to the formation of a denser, less fluid interfacial film in the emulsion, inhibiting flocculation and coalescence and maintaining emulsion stability. Notably, new peaks at 28°, 32°, and 45° (2θ) confirmed the formation of a unique co-assembled structure. Thus, these findings suggest WCM nanoparticles exhibited intermediate characteristics, reflecting compatibility between WPs and CMC to form a stable co-assembled structure.

### 3.4 Surface morphology of WCM nanoparticles

**Fig. 4:**
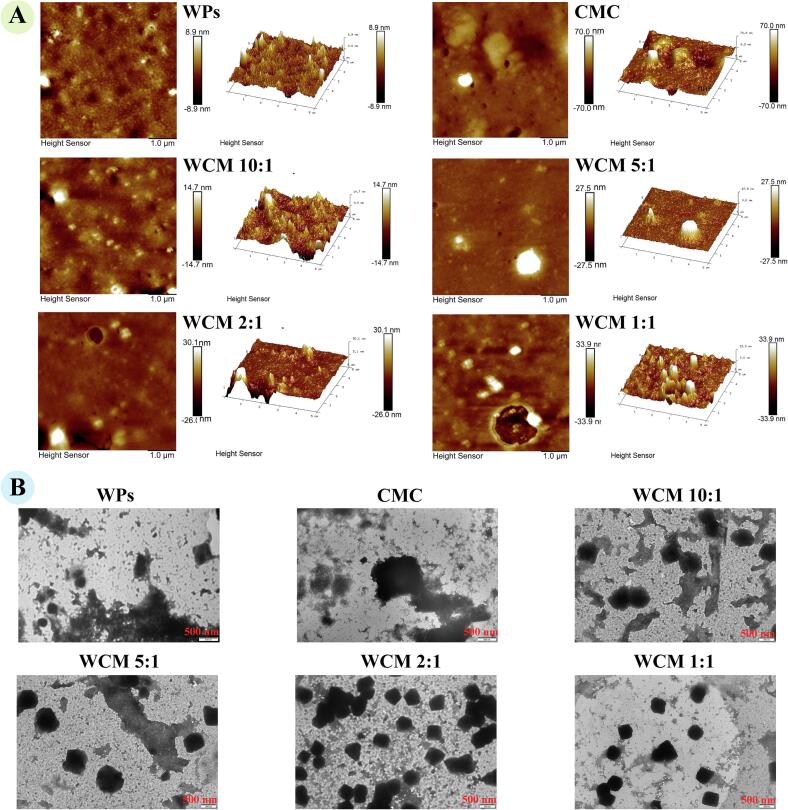
Surface morphology of WPs, CMC, WCM 10:1, WCM 5:1, WCM 2:1 and WCM 1:1; (A) AFM showed the surface roughness and 3D morphology of the samples with a scale bar of 1.0 μm; (B) TEM displayed representative micrographs with a scale bar of 500 nm.

The surface morphology and properties of WCM nanoparticles were characterized using AFM, with results shown in Fig. 4-A. WPs exhibited a characteristic spherical morphology typical of proteins, while CMC formed supramolecular protofibrils that assembled into irregular, network-like structures. A certain amount of CMC can modify WPs, transforming their morphology from protein spheres to networks. As the WPs: CMC ratio decreased, the height of WCM nanoparticles correspondingly increased, indicating the formation of larger particle clusters through their co-assembly. This increase in height and roughness is typically associated with cluster growth/bridging, confirming that the CMC fiber network can bridge adjacent WP particles (Feng et al., 2024). The WCM nanoparticles predominantly retained the spherical morphology of WPs, suggesting that proteins served as the primary structural framework, with CMC interacting *via* specific intermolecular forces. This observation aligns with findings by Li et al. (2025), who reported that egg white proteins co-assembled with CMC formed anisotropic, bead-like supramolecular fiber networks. WCM 2:1 nanoparticles displayed a regular elliptical shape with uniform dispersion, whereas WCM 1:1 nanoparticles were larger and exhibited agglomeration. Thus, at a WPs: CMC mass ratio of 2:1, strong interactions between WPs and CMC facilitated the formation of stable nanocomplexes. Appropriate bridging between CMC and WPs can form a dense structure, which contributes to better dispersion of WCM nanoparticles in the emulsion and the formation of a stable interface layer. However, excessive bridging causes them to agglomerate, weakening their functionality. In conclusion, these changes affect the encapsulation efficiency, storage stability, and release properties of WCM nanoparticles for active substances.

The morphology of WCM nanoparticles observed using TEM further elucidated the microstructural changes before and after the co-assembly of WPs (Fig. 4-B). WPs displayed a spherical structure, while CMC appeared as a fiber-interwoven mesh. WCM 10:1 nanoparticles exhibited a loose, ring-like outer edge, indicative of a spherical core-shell morphology resulting from WPs-CMC co-assembly. In complexes of hydrophobic proteins and hydrophilic polysaccharides, hydrophobic groups typically form an internal core, while hydrophilic segments are bridged on the surface to create a surface shell (Bayliss & Schmidt, 2023; Feng et al., 2024; Li et al., 2025). With decreasing WPs: CMC ratio, WCM nanoparticles developed a more pronounced spherical profile and enhanced structural homogeneity. These findings suggest that, through a surface patch effect, positively charged WPs regions bind to anionic CMC groups during pH adjustment, forming chain-bead nanostructures (Hayati et al., 2018b). Moreover, an optimal CMC content enables robust and cohesive interactions with WPs, yielding a stable composite structure (Hayati & Gutiérrez, 2018a). A stable and uniform structure enables WCM nanoparticles to rapidly emulsify and form stable emulsions, thereby firmly encapsulating active substances. Therefore, the degree of combination between WPs and CMC has a critical impact on the functionality of WCM nanoparticles.

### 3.5 Emulsification properties and stability of WCM nanoparticles

**Fig. 5:**
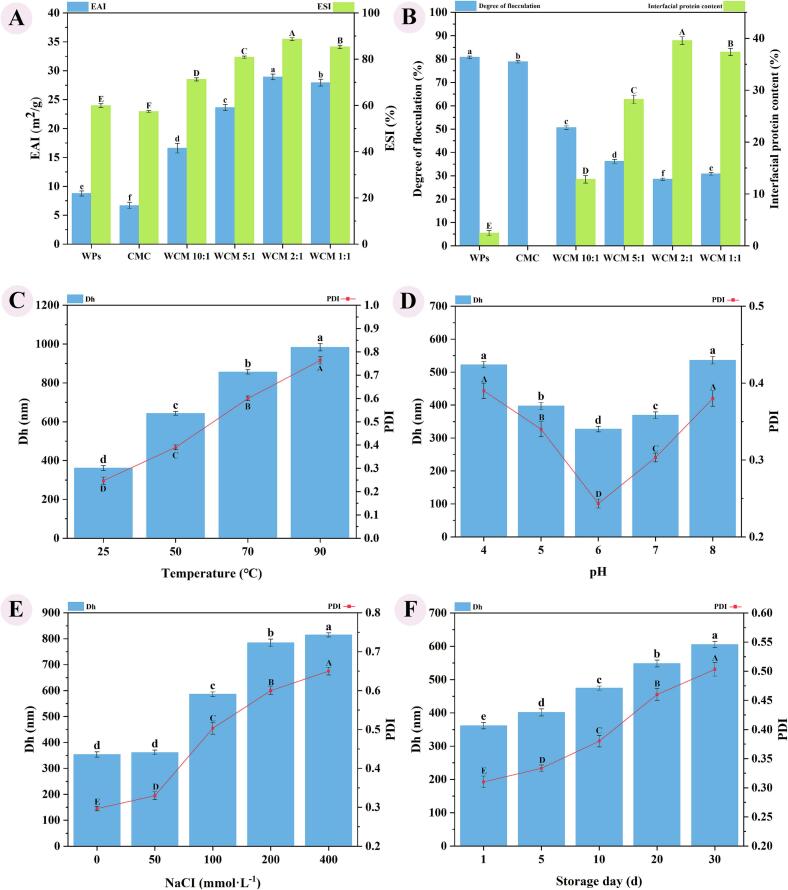
Emulsification properties and stability of WCM nanoparticles; (A) EAI and ESI; (B) Degree of flocculation and interfacial protein content; Particle size (Dh), and polydispersity index (PDI) were measured to evaluate (C) temperature stability, (D) pH stability, (E) salt stability, and (F) storage properties of WCM 2:1 nanoparticles. Mean ± standard deviation is represented (*n* = 3). Different letters indicate significant differences with control (a-e; A-E).

The emulsification properties and stability of WCM nanoparticles were evaluated, with results shown in Fig. 5. WCM nanoparticles (16.60–28.93 m^2^/g, 59.91 min) exhibited significantly higher EAI and ESI compared to WPs (8.75 m^2^/g, 59.91 min) and CMC (6.67 m^2^/g, 57.33 min), indicating their potential for emulsion applications (Fig. 5-A). EAI and ESI of WCM nanoparticles are comparable to most protein-polysaccharides (Han et al., 2024), such as whey protein isolate-dandelion polysaccharide complex (32.61 m^2^/g,42.58 min), indicating that they can be applied to food emulsion systems. As the WPs: CMC ratio decreased, EAI and ESI of WCM emulsions showed an upward trend, attributed to enhanced protein solubility and co-assembly effects (Fig. 5-A). Moreover, the incorporation of hydrophilic hydroxyl groups from CMC improved the hydrophilic-lipophilic balance, enhancing emulsification properties. Additionally, during emulsification, the protein structure extended to form a stable interfacial layer encapsulating the oil phase. However, the EAI and ESI of WCM 1:1 emulsion started to decrease likely due to excessive CMC, which increased droplet dispersibility and electrostatic repulsion, distancing protein particles from the oil-water interface.

WCM emulsions demonstrated superior anti-flocculation compared to WPs and CMC, resulting from a cohesive interfacial layer stabilized by non-covalent interactions, including electrostatic, hydrophobic, hydrogen bonding, and Van der Waals forces (Fig. 5-B). The protein-polysaccharide interactions formed a thicker interfacial layer at the oil-water surface, providing steric repulsion that prevented droplet coalescence and aggregation of oil droplets (Luo & Wei, 2023). In addition, higher interfacial protein content provided additional binding sites, enabling tighter adhesion to the oil-water interface, thereby enhancing emulsion stability (Fig. 5-B). WCM nanoparticles, formed through WPs-CMC co-assembly, adsorbed at the oil-water interface, creating a dense three-dimensional mesh structure that reduced interfacial tension.

Stable emulsions typically exhibit smaller droplet sizes and uniform distribution. Given that emulsion-based food products are often exposed to varying temperature, pH, and ionic strength during processing, the effects of these factors on the stability of the WCM 2:1 emulsion were investigated (Fig. 5). With increasing temperature, the particle size increased from 360.91 nm to 643.20 nm with a PDI of 0.39 at 50 °C, indicating that the emulsion was relatively stable (Fig. 5-C). Further temperature increases led to protein denaturation and degradation of co-assembled structures, disrupting the adsorbed interfacial membrane and causing emulsion destabilization. These results suggest that the WCM 2:1 emulsion resist deformation at moderate temperatures (< 50 °C), but elevated temperatures increase droplet size and compromise stability.

The WCM 2:1 emulsion demonstrated good stability at pH 5.0–7.0 (Fig. 5-D), with optimal performance at pH 6.0 (Dh = 326.97 nm, PDI = 0.24). Moreover, extreme alkaline environments made emulsion unstable, leading to increased emulsion droplet size and reduced stability. Since CMC is very stable in this pH range, it is mainly the extreme acid-base conditions that alter the structure and properties of WPs, thereby weakening their co-assembly and reducing their interfacial packing density. Strongly acidic conditions cause the hydrogen and ionic bonds of proteins to break, while highly alkaline can lead to protein unfolding and aggregation, exposing hydrophobic patches. These all disrupt the stability of the interfacial membrane. As shown in Fig. 5-E, the addition of NaCl significantly increased the particle size (Dh) of the WCM 2:1 emulsion, likely due to enhanced electrostatic repulsion, which promoted droplet-to-droplet repulsion and flocculation. Although low salt ions minimally affect the structures of WPs, CMC, and WCM 2:1 nanoparticles, they can influence the interfacial thickness, structure, and composition mainly through charge effects, thereby altering emulsion stability. However, high salt concentrations (>100 mM) cause further protein conformational changes that promote aggregation, resulting in larger droplet sizes and phase separation. This can be due to the fact that Na^+^ can interact with the CMC's carboxyl groups to alter its hydration state, causing thinner boundary films that are prone to coalescence. At low NaCl concentrations (<50 mM), the WCM 2:1 emulsion exhibited significant stability, as nanoparticles could mitigate electrostatic repulsion between droplets, stabilizing the interfacial film.

Furthermore, storage stability, one of the key quality parameters for emulsions, directly influences their industrial applicability. The WCM 2:1 emulsion showed robust stability, with gradual changes in Dh and PDI over time (Fig. 5-F). Even after 30 days of storage, the Dh was 605.05 nm (<1000 nm), and the PDI was 0.50. These findings indicate that WPs co-assembled with CMC form composite nanoparticles suitable for applications in emulsified food products.

### 3.6 Emulsion micromorphology

**Fig. 6:**
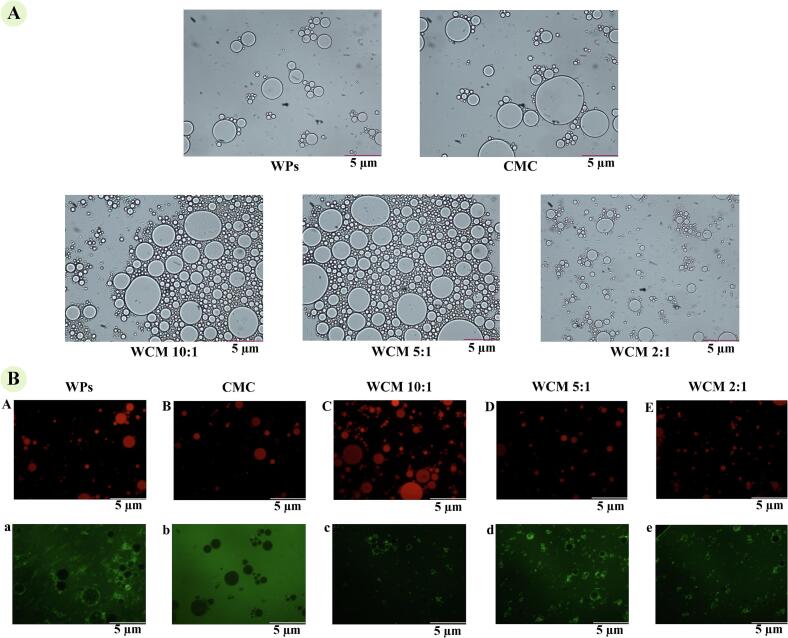
Optical and fluorescence microscope observation of emulsion. (A) Optical microscopy micrographs of WPs, CMC, WCM 10:1, WCM 5:1, and WCM 2:1 emulsion was displayed with a scale bar of 5.0 μm; (B) the A-E micrographs at excitation wavelengths of 488 nm (Nile Red) showed the oil staining shape, and a-e micrographs at excitation wavelengths of 633 nm (Nile blue) displayed the protein staining form. Scale bar of 5.0 μm. (For interpretation of the references to colour in this figure legend, the reader is referred to the web version of this article.)

The micromorphology of emulsions was examined using optical microscopy, with results shown in Fig. 6-A. Emulsion droplets formed by WPs were smaller than those formed by CMC, while WCM emulsions (WCM 10:1 and WCM 5:1) contained a significantly higher number of droplets. Notably, WCM 2:1 emulsion exhibited smaller, uniformly distributed droplets, indicating strong binding between WPs and CMC at this ratio, which enhanced the compatibility of composite nanoparticles with their emulsification properties.

Laser confocal microscopy was employed to investigate the distribution of proteins and oil droplets (Fig. 6-B). WPs emulsion displayed larger droplet shape with proteins adsorbed onto the oil droplet surfaces, similar to CMC emulsions (Fig. 6-B), indicating that WPs and CMC corresponded to general emulsification properties. In WCM 10:1 emulsion, more particles were adsorbed onto oil droplets, but the droplets remained relatively large. Despite the enhanced emulsification performance of WPs-CMC composite nanoparticles, insufficient interactions between droplets hindered uniform distribution, resulting in larger droplets throughout the system. This was influenced by the particle distribution, as well as the formation and stabilization mechanisms of the emulsion. As the WPs: CMC ratio decreased, WCM emulsion lipid droplets became smaller and more homogeneously distributed, with a progressive increase in adsorbed interfacial proteins. The WCM 2:1 emulsion exhibited optimal morphology, attributed to composite nanoparticles restricting the movement of proteins and lipid droplets through intermolecular interactions and steric hindrance, leading to a more ordered distribution (Yang et al., 2024). Furthermore, WCM nanoparticles demonstrated enhanced hydration, hydrophilicity, and lipophilicity, which collectively contributed to improved emulsion stability.

### 3.7 Emulsion loading capacity of WCM nanoparticles

**Fig. 7:**
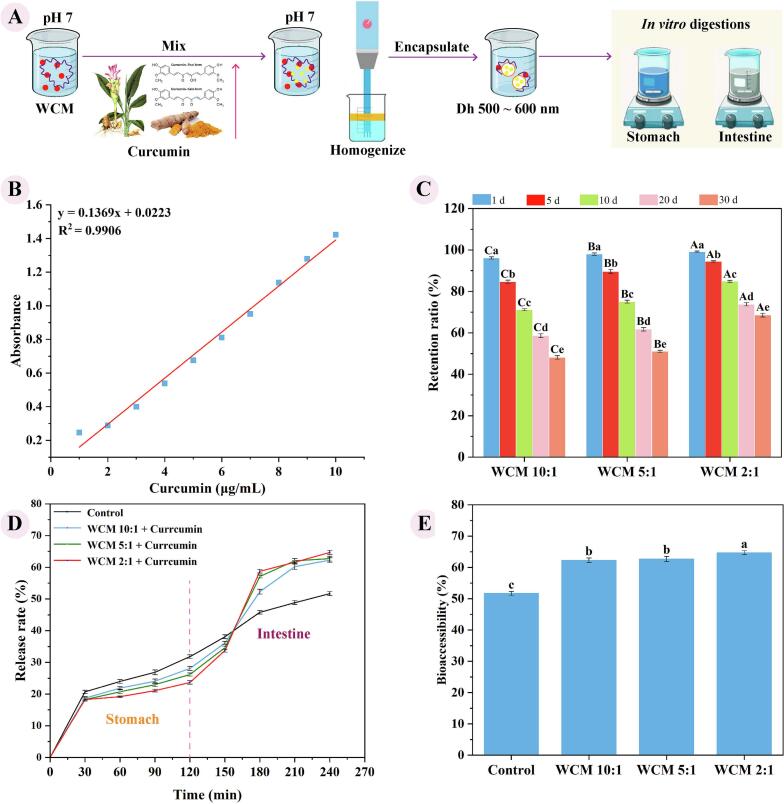
Effects of WCM nanoparticle emulsion on the loading capacity and bioaccessibility of curcumin; (A) Schematic diagram of loading curcumin; (B) Standard curve of curcumin in ethanol (1–10 μg/mL); (C) Curcumin retention rate in the WCM nanoparticles emulsions after 1, 5, 10, 20, and 30 days, where capital letters (A-C) indicate the mean values significantly different between three WCM nanoparticles within the same day (p < 0.05), and lowercase letters (a-e) indicate significance between different days within each WCM nanoparticle (p < 0.05); (D) Release rate of curcumin *in vitro* simulated gastrointestinal digestion; (E) Bioaccessibility, where the a-c indicated the mean values were significantly different (p < 0.05).

WCM nanoparticles were utilized to prepare emulsions *via* high-speed homogenization, incorporating curcumin solubilized in linseed oil. The co-assembled structure of WPs and CMC provided hydrophobic cavities and binding sites, facilitating the encapsulation of hydrophobic curcumin (Fig. 7-A). A standard curve for curcumin in anhydrous ethanol (1–10 μg/mL) was established (Fig. 7-B) to quantify the loading capacity of WCM nanoparticles and assess subsequent release during *in vitro* digestion. The effect of storage time on curcumin retention in WCM nanoparticle emulsions is shown in Fig. 7-C. Curcumin retention decreased over time but remained above 70 % after 10 days of storage. Notably, retention increased with lower WPs: CMC ratio, reaching 84.88 % after 10 days at a ratio of 2:1. This high retention was attributed to the formation of a dense interfacial film by WCM nanoparticles at the oil-water interface under room temperature conditions, contributing to loading and protecting curcumin. Additionally, the interactions between WPs and CMC were critical for curcumin encapsulation, sustaining a complex network structure around oil droplets that effectively limited the diffusion of oxygen, oxidants, and free radicals, thereby reducing curcumin degradation. Compared with zein-whey protein isolate-pectin composite nanoparticles (encapsulation efficiency 60.8–87.5 %), WCM nanoparticles emulsion exhibits excellent encapsulation performance, but is inferior to some protein-polysaccharide systems (up to 95 %) (Gu, Li, Jiang, Chang, & Wu, 2024). Therefore, the emulsion formed by WCM nanoparticles can effectively encapsulate curcumin, which is mainly incorporated in the hydrophobic region of proteins, enabling it to be stabilized.

### 3.8 Digestion and release properties

The release rates of curcumin during *in vitro* simulated digestion are shown in Fig. 7-D. During the gastric digestion phase (0–120 min), the release rate of curcumin from WCM nanoparticle emulsions was consistently lower than that of the control group, indicating effective protection against degradation of the active compound. As the WPs: CMC ratio decreased, the WCM 2:1 emulsion exhibited the most pronounced protective effect, consistent with its previously described compact and stable co-assembled structure. This is precisely attributed to the fact that WCM 2:1 nanoparticles form a tight interface network at the emulsion interface, making them relatively more effective in protecting proteins from hydrolysis and aggregation at this stage (Wang et al., 2023). In addition, the stable bridging structure of WCM nanoparticles reduce the possibility of encapsulated curcumin being exposed to acidic gastric juices and oxidative degradation. Therefore, the protective capacity of WCM nanoparticles limit the premature release of curcumin during gastric digestion, enhancing its stability. In the intestinal digestion phase (120–240 min), the curcumin release rate accelerated and was higher than that of the control group, with the WCM 2:1 emulsion demonstrating superior performance. Trypsin in the intestinal fluid specifically targets protein molecules at the emulsion interface, compromising the integrity of the interfacial membrane and enabling rapid decomposition and release of curcumin embedded in the droplets. Additionally, bile salts and peptides formed composite micelles with released curcumin, facilitating its uptake, transport, and absorption in the small intestine. Therefore, WCM nanoparticles emulsion enables rapid release of curcumin in the intestine, further distributing it more effectively into mixed micelles.

As shown in Fig. 7-E, the bioaccessibility of curcumin in the emulsion systems was 62.25 % (WCM 10:1), 62.70 % (WCM 5:1), and 64.68 % (WCM 2:1) respectively. These values were significantly higher than those of control group (51.69 %) (*p* < 0.05). Conversely, in this study pH cycling was only preliminarily applied to promote the co-assembly of WPs and CMC, without optimizing the WCM nanoparticles, resulting in their incomplete functionality. Moreover, the mechanism by which active ingredients could be loaded into WCM nanoparticles and their pharmacokinetics *in vivo* are unknown. Still, these results demonstrate that WPs and the CMC component of WCM nanoparticles synergistically stabilize curcumin, enhancing its stability. Moreover, smaller droplet sizes increased the contact surface area with lipase, accelerating lipolysis and enabling greater curcumin dissolution into micelles for intestinal release. Therefore, protein and polysaccharide co-assembled nanoparticle emulsions can ensure that curcumin is protected from early degradation in gastric juice, enabling it to dissolve effectively in mixed micelles during intestinal digestion, which maximizes its bioaccessibility and potential absorption rate. The digestive release characteristics of the WCM nanoparticle system are consistent with most conventional protein-polysaccharide nanocarriers, improving the bioavailability of curcumin, but it is inferior to some advanced delivery systems (Jiang et al., 2023). In conclusion, the WCM nanoparticle emulsion system effectively improves the bioaccessibility of curcumin.

## 4 Conclusion

pH cycling altered the folding behavior of WPs, exposing binding sites to interact with CMC and form stable WCM 2:1 nanoparticles with Dh 832.40 nm and PDI 0.38. It was the CMC that inhibited protein refolding during the neutralization process from pH 12.0 to 7.0, promoting a hydrophilic conformation through co-assembly with WPs, thereby increasing its protein solubility to 75.34 %. Hydrophobic and electrostatic interactions were the primary forces involved in co-assembly process, and the formed WCM nanoparticles showed stronger hydrogen bonding than WPs as demonstrated by FTIR spectroscopy. Morphological studies have shown that appropriate bridging between CMC and WPs can form a dense fibrous network structure, exhibiting a core-shell morphology. XRD findings indicated that the fiber network of CMC rendered the structure of WCM nanoparticles more orderly and moderately increased its crystallinity, thereby enhancing its emulsification function. WCM nanoparticles exhibited excellent emulsification properties, with the CMC component facilitating their adsorption at the oil-water interface, while the hydrophobic groups of WPs played a key role in maintaining emulsion stability. These WCM nanoparticle emulsions effectively encapsulated curcumin, with *in vitro* digestion studies demonstrating that WCM 2:1 enhanced curcumin bioaccessibility (64.68 %). In the future, it will be necessary to modify WCM nanoparticles to improve their functionality and demonstrate their edible safety. Overall, this study addresses the challenges of utilizing WPs in emulsions, provides new insights into the mechanism of protein-polysaccharide interactions, broadening their application in functional delivery systems.

## CRediT authorship contribution statement

**Pengren Zou:** Writing – original draft, Methodology, Investigation, Formal analysis. **Celia Costas:** Writing – review & editing, Supervision, Methodology, Investigation, Formal analysis, Conceptualization. **Susan Jyakhwo:** Writing – review & editing. **Yihao Luo:** Writing – original draft, Methodology, Investigation. **Li Luo:** Writing – original draft, Methodology, Investigation. **Zhaojun Wei:** Writing – review & editing, Supervision, Project administration. **Paz Otero:** Writing – review & editing, Supervision, Project administration, Methodology, Investigation, Funding acquisition, Formal analysis, Conceptualization.

## Declaration of competing interest

The authors declare that they have no known competing financial interests or personal relationships that could have appeared to influence the work reported in this paper.

## Data Availability

Data will be made available on request.
